# The prevalence, associated factors, clinical impact, and state of diagnosis of delirium in palliative care patients

**DOI:** 10.1007/s00520-021-06367-7

**Published:** 2021-07-02

**Authors:** Watanachai Klankluang, Sasima Tongsai, Chairat Sriphirom, Arunotai Siriussawakul, Pratamaporn Chanthong, Supakarn Tayjasanant

**Affiliations:** 1 Siriraj Palliative Care Center, Faculty of Medicine Siriraj Hospital, Mahidol University, 2 Wanglang Rd. Bangkoknoi, Bangkok, 10700 Thailand; 2 Office for Research and Development, Faculty of Medicine Siriraj Hospital, Mahidol University, Bangkok, Thailand; 3 Department of Anesthesiology, Faculty of Medicine Siriraj Hospital, Mahidol University, Bangkok, Thailand; 4 Integrated Perioperative Geriatric Excellent Research Center, Faculty of Medicine Siriraj Hospital, Mahidol University, Bangkok, Thailand

**Keywords:** Delirium, Supportive care, Prevalence, Survival, Cancer, Associated factor

## Abstract

**Purpose:**

The aim of this study is to establish the prevalence, associated factors, and clinical impact of delirium in newly referred palliative care patients and the percentage of delirium diagnoses missed by primary medical teams.

**Methods:**

Newly referred palliative care patients were evaluated and were reviewed for possible associated factors of delirium. Univariable and multivariable analysis were used to identify associated factors. Median overall survival and survival curves were analyzed. The percentage of missed diagnosis in IPD patients was identified.

**Results:**

We included 350 palliative care patients. Nearly all patients had cancer diagnosis (96.6%). The overall prevalence of delirium was 44.0%. The independent associated factors of delirium were age ≥ 63 years (adjusted odds ratio [aOR], 7.0; 95% CI, 2.2–22.9), palliative performance scale ≤ 20% (aOR, 54.5; 95% CI, 13.1–228.0), brain metastasis (aOR, 15.6; 95% CI, 3.7–66.7), urinary tract infection (aOR, 18.8; 95% CI, 4.7–75.5), sepsis (aOR, 59.0; 95% CI, 4.4–797.8), hyponatremia (aOR, 8.8; 95% CI, 2.6–29.8), and hypercalcemia (not applicable). Interestingly, opioids and benzodiazepines were not associated with delirium. Delirious patients had significantly shorter survival (median survival 11 days). Delirium diagnoses were missed for 76.1%.

**Conclusion:**

Nearly half of the palliative care patients had delirium, which was associated with noticeably short survivals. We identified the independent factors associated with the delirium. Despite having a remarkably high prevalence rate and being a well-known poor prognostic factor, there was still a very high rate of missed delirium diagnoses. Effective, routine, delirium screening of palliative care patients needs to be emphasized.

## Introduction

Delirium is common in the palliative care population [1], with reported prevalences of 13–93% varying with multiple factors, such as comorbidities, study setting, and how far along patients are in their disease trajectories [2–4]. Delirium is characterized by acute disturbances to awareness, attention, and cognitive functions [5]. It is also associated with increased morbidity and mortality rates, higher symptom expression, prolonged hospitalization, negative financial consequences, and impediments to communication, all of which cause distress to patients and their families [6–9]. Unfortunately, delirium is frequently misdiagnosed for a range of reasons, including medical personnel’s lack of awareness and knowledge, the fluctuating nature of delirium symptoms and their resemblance to those of other psychiatric disorders, and inadequate regular screening for delirium [10–13].

As to a multifactorial model, delirium is the result of a combination of predisposing and precipitating factors [14]. However, the factors that are common to delirious palliative care patients have not been well characterized, with structured evidence of the predisposing and precipitating factors of delirium in palliative care patients being sparse [2, 4]. The most frequently occurring predisposing factors are older age, reduced performance status, and brain metastasis [3, 15–17], while the most prevalent precipitating factors are opioids, other psychoactive medications, electrolyte imbalance, and infections [18–20]. Comprehensive characterization of the causes of delirium could promote prompt investigations and appropriate interventions [21].

Our primary objectives were to identify the prevalence and associated factors of delirium among palliative care patients. The secondary objectives were to (1) examine the clinical impact of delirium on survival time and length of hospital stay and (2) determine the percentage of delirium diagnoses missed by the primary medical teams (PMTs) in a palliative care setting.

## Materials and methods

### Study population

The protocol was approved by the institution review board of the Faculty of Medicine Siriraj Hospital, Mahidol University; Reference number Si 421/2017. The participants were patients aged 18 years or older with advanced cancer or major organ failure who had been referred to the outpatient or inpatient department (OPD or IPD) of the palliative care center. However, due to ethical consideration, patients were excluded if the psychiatrist confirmed that they had a persistent symptom score of 7 or more in any item of the Edmonton Symptom Assessment System. Furthermore, patients diagnosed with dementia or mental retardation, had coma, or had a communication difficulty caused by an endotracheal intubation or a tracheostomy were excluded. Written informed consents were obtained from the patients or their proxies.

The participants were assessed by a psychiatrist using the Diagnostic and Statistical Manual of Mental Disorders, Fifth Edition criteria for delirium [22] via a semi-structured interview. If delirium was diagnosed, the subtype (hyperactive, hypoactive, and mixed) was identified according to the psychomotor activity exhibited by the patient. All patients were reviewed for potential factors associated with delirium by reviewing their history of illness, treatment-related factors, physical examination findings, and laboratory investigations. All of them were followed up until death and the prevalence rate of delirium in patients with survival time 31–60, 15–30, 4–14, and 1–3 days were calculated.

To facilitate the analysis of the diagnoses made by the PMTs, the patients in the IPD group were grouped into those who were (1) referred for other symptoms and were not found to have delirium (“no delirium”); (2) referred for other symptoms, but were found to have delirium (“missed diagnosis”); (3) correctly diagnosed with delirium by the PMTs (“correct diagnosis”); and (4) referred with suspected delirium, but were not found to have a diagnosis of delirium (“incorrect diagnosis”). We included only the IPD patients in this analysis because they were seen within 24 h of referral. In contrast, the OPD patients had a lag time between their referral and appointment dates; they could have developed delirium during this period, which would have affected the correctness of their diagnoses.

### Statistical analysis

The main study objectives were to determine the prevalence and the associated factors of delirium in patients referred to the palliative care center. Surveillance data from the Siriraj Palliative Care Center in 2016 demonstrated a delirium prevalence of 35%; this figure was used to calculate the sample size of the present study. With an allowable error of 5% at a 95% confidence interval (CI), nQuery Advisor version 5.0 yielded an evaluable sample size of 350 patients.

A post hoc power analysis was conducted to verify the adequacy of the sample size for multiple logistic regression analysis using the program G*Power (version 3.1.9.2) [23]. We considered a model with a single predictor (age ≥ 63 years), which was binomially distributed and identified as the weakest independent factor associated with delirium by our multivariable analysis. Using that, with a base rate of age 63 ≥ years to be delirium of 0.53, a two-tailed z test, an event rate under H_0_ of π_1_ = 0.56, an adjusted odds ratio for the predictor (age ≥ 63 years) of 7.044, and a total sample size of 350, the proportion of variance of age ≥ 63 years was explained by 7 independent predictors of delirium in the model of 11%; we therefore needed an “R2 other X” of 0.11. When using the Demidenko method for sample size calculation for logistic regression [24] (with variance correction), the post hoc power analysis showed a power of 0.9999, which suggested the study had adequate power to detect an association between 8 independent predictors and delirium.

Data were prepared and analyzed using PASW Statistics for Windows (version 18.0; SPSS Inc., Chicago, Ill., USA). The quantitative data were expressed as means and standard deviations, or as medians and interquartile ranges, as appropriate, while the qualitative data were summarized with frequencies and percentages. Normality of data distribution was verified with the Shapiro–Wilk test. To compare the demographics, clinical characteristics (comorbidities, precipitating variables, and medications used), and clinical impacts of the delirium and non-delirium patients, an unpaired t-test or the Mann–Whitney U test was used for the quantitative data, whereas Pearson’s chi-squared test, Yates’ continuity correction, or Fisher’s exact test was used for the qualitative data, as appropriate. A receiver operating characteristic curve was used to determine the optimal cutoff value of age and palliative performance scale (PPS) for the prediction of delirium. A univariable analysis was performed to identify the factors associated with delirium; the variables with a P value of < 0.05 were subsequently included in a backward multiple logistic regression analysis to identify the independent predictors of delirium. Odds ratios (ORs) were used to evaluate the strength and direction of the association between the factors and delirium.

Median overall survival and overall survival curves were analyzed using the Kaplan–Meier method. The survival times were calculated from the date of consultation to the date of death, while the endpoint was the 1-year follow-up; the patients who were still alive at the end of the 1-year observation period were censored. A log-rank test was performed to compare the overall survival curves of the delirium and non-delirium groups. All tests of significance were two-tailed, and *P* < 0.05 was considered statistically significant.

## Results

Between July 2017 and October 2018, 743 newly referred, palliative care patients were consecutively recruited. However, 393 of those were not enrolled. Sixty-three were excluded because they had a persistent symptom score of 7 or more in the Edmonton Symptom Assessment System (28 patients with pain, 25 with dyspnea, and 10 with depression). A further 43 patients were excluded due to having coma (28 patients), dementia (10), or communication difficulties resulting from endotracheal intubation or tracheostomy (5). Moreover, the consent of 33 patients to be involved in the research was not able to be obtained because of their altered state of consciousness and the absence of their proxies. The 254 remaining patients declined to participate in the study.

In all, 350 palliative care patients were enrolled. The prevalences of delirium in all 350 patients, the 140 OPD patients, and the 210 IPD patients were 44.0%, 26.4%, and 55.7%, respectively. The prevalences of delirium in patients with survival time 31–60, 15–30, 4–14, and 1–3 days were 32.7% (17/52), 44.6% (25/56), 72.4% (55/76), and 88.9% (32/36), respectively. In the delirium group, most patients had the mixed subtype (53.9%), followed by the hypoactive (38.3%) and hyperactive (7.8%) subtypes.

**Fig. 1:**
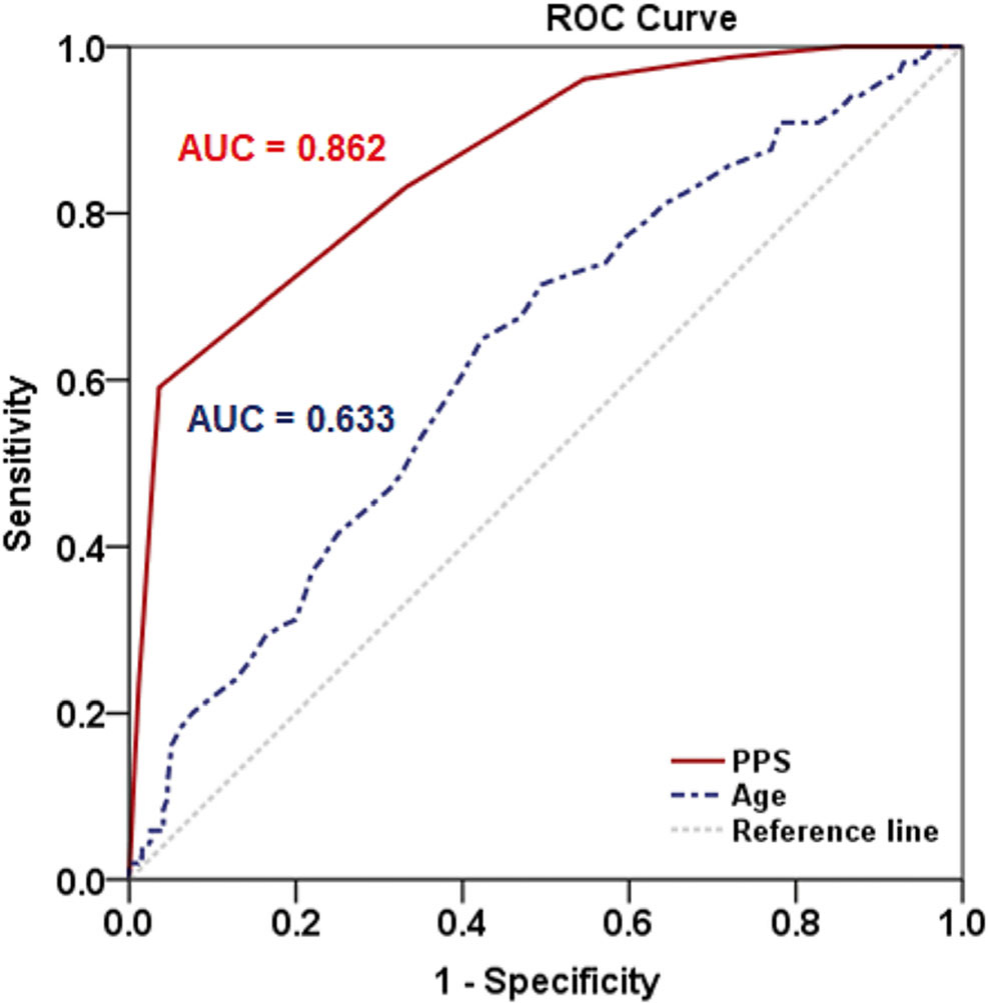
The area under the receiver operating characteristic curve (AUC) of age and PPS for predicting delirium. The optimal cut-off value for age was ≥ 63 years, AUC = 0.633 (95% CI: 0.575–0.691) sensitivity = 67.5% (95% CI 59.8–74.4%), specificity = 53.1% (95% CI 46.1–59.9%), and accuracy = 59.4% (95% CI 54.2–64.4%). The optimal cut-off value for PPS was ≤ 20%, AUC = 0.862 (95% CI: 0.825–0.900), sensitivity = 59.1% (95% CI 51.2–66.5%), specificity = 96.4% (95% CI 92.8–98.3%), and accuracy = 80.0% (95% CI 75.5–83.9%). Abbreviation: *CI* confidence interval

The results of the univariable analysis of the demographics, clinical characteristics, medication usage, and clinical impacts are detailed in Table 1. We found that age and the PPS values of the delirium and non-delirium groups had statistically significant differences both (*P* values were < 0.001). The optimal cut-off values of age and PPS for delirium were then calculated. The areas under receiver operating characteristic curves, sensitivities, specificities, and accuracies of age and PPS are presented in Fig. 1. The results of the subsequent multivariable analysis of the independent predictors of overall delirium are listed in Table 2. The unadjusted OR and adjusted OR of hypercalcemia and patients with no apparent precipitating cause were not applicable because there was no event in the non-delirium group for these factors.

**Table 1:** Demographics, clinical characteristics, and delirium impacts of palliative care patients (n = 350)

Factors	Delirium (n = 154)	Non-delirium (n = 196)	P value	
Age, mean ± SD, years Age ≥ 63, n (%)	67.8 ± 12.6 104 (67.5)	61.8 ± 13.5 92 (46.9)	** < .001**	
Sex, no. of M/F, n (%)	83:71 (53.9:46.1)	94:102 (48.0:52.0)	.320	
Setting, n (%)			** < .001**	
Outpatient	37 (24.0)	103 (52.55)		
Inpatient	117 (76.0)	93 (47.45)		
Unemployed, n (%)	118 (76.6)	125 (63.8)	**.013**	
Palliative performance scale, median (IQR) Palliative performance scale ≤ 20%, n (%)	20 (20–30) 91 (59.1)	40 (30–60) 7 (3.6)	** < .001**	
Cancer diagnosis, n (%)	145 (94.2)	193 (98.5)	.057	
Organ site of cancer, n (%)			.810	
Genitourinary	19 (13.1)	26 (13.5)		
Gastrointestinal tract	31 (21.4)	49 (25.4)		
Lung	28 (19.3)	30 (15.5)		
Liver and bile ducts	17 (11.7)	23 (11.9)		
Pancreas	9 (6.2)	11 (5.7)		
Head and neck	17 (11.7)	17 (8.8)		
Breast	12 (8.3)	16 (8.3)		
Unknown primary	5 (3.4)	4 (2.1)		
Others	7 (4.8)	17 (8.8)		
Cancer type, n (%)			.170	
Locally advanced	40 (26.0)	56 (28.6)		
Metastatic	100 (64.9)	126 (64.3)		
Recurrent	4 (2.6)	10 (5.1)		
Relapsed	1 (0.6)	1 (0.5)		
Distant metastasis, n (%)				
Liver	39 (36.8)	40 (30.5)	.380	
Lung	31 (29.2)	42 (32.1)	.745	
Pleural	7 (6.6)	17 (13.0)	.161	
Bone	46 (43.4)	46 (35.1)	.243	
Brain	22 (20.8)	9 (6.9)	**.003**	
Lymph node	12 (11.3)	13 (9.9)	.892	
Peritoneal	5 (4.7)	11 (8.4)	.389	
Others	13 (12.3)	19 (14.5)	.756	
Comorbidities, n (%)				
Diabetes mellitus	37 (24.0)	34 (17.3)	.159	
Hypertension	65 (42.2)	56 (28.6)	**.011**	
Dyslipidemia	38 (24.7)	26 (13.3)	**.009**	
Cerebrovascular accident	9 (5.8)	3 (1.5)	.057	
Chronic heart disease	18 (11.7)	9 (4.6)	**.025**	
Chronic hepatic disease	19 (12.3)	12 (6.1)	.065	
Chronic kidney disease	30 (19.5)	7 (3.6)	** < .001**	
Other psychiatric disorder	3 (1.9)	5 (2.6)	.989	
Precipitating, n (%)				
Dehydration	49 (31.8)	17 (8.7)	** < .001**	
Pneumonia	21 (13.6)	7 (3.6)	**.001**	
Urinary tract infection	29 (18.8)	9 (4.6)	** < .001**	
Sepsis	16 (10.4)	1 (0.5)	** < .001**	
Acute kidney injury	34 (22.1)	7 (3.6)	** < .001**	
Hypercalcemia	27 (17.5)	0 (0.0)	** < .001**	
Hyponatremia	44 (28.6)	16 (8.2)	** < .001**	
Hypernatremia	5 (3.2)	5 (2.6)	.948	
Uremia	5 (5)	0 (0.0)	**.037**	
Anemia	30 (19.5)	24 (12.2)	.087	
No apparent precipitating cause	28 (18.2)	0 (0.0)	** < .001**	
Morphine equivalence daily dose, median (IQR)	6.8 (0–30)	15.5 (0–30)	.382	
Benzodiazepine, n (%)	13 (8.4)	31 (15.8)	.057	
Impact, median (IQR)				
Length of stay, days	5 (2–8)	8 (3–16)	** < .001**	
Number of emergency visits	0 (0–1)	0 (0–1)	.229	
Number of hospital admissions	1 (1–1)	1 (0–1)	.221	

P value <0.05 indicates statistical significance

**Table 2:** Independent predictors of delirium (n = 350)

Factors	Crude OR (95% CI)	P value	Adjusted OR^a^ (95% CI)	P value
Age ≥ 63	2.3 (1.5–3.6)	< .001	7.0 (2.2–22.9)	.001
Palliative Performance Scale ≤ 20%	39.0 (17.2–88.5)	< .001	54.5 (13.1–228.0)	< .001
Brain metastasis	3.6 (1.6–8.1)	.003	15.6 (3.7–66.7)	< .001
Urinary tract infection	4.8 (2.2–10.5)	< .001	18.8 (4.7–75.5)	< .001
Sepsis	22.6 (3.0–172.5)	.003	59.0 (4.4–797.8)	.002
Hyponatremia	4.5 (2.4–8.4)	< .001	8.8 (2.6–29.8)	< .001
Hypercalcemia	NA	NA	NA	NA
No apparent precipitating cause	NA	NA	NA	NA

*NA,* not applicable

^a^Analysis adjusted for factors listed in Table 2; these comprised setting, unemployed, hypertension, dyslipidemia, chronic heart disease, chronic kidney disease, pneumonia, acute kidney injury, hypernatremia, and uremia. The eight factors listed in Table 2 were retained in the final model that used a backward multiple binary logistic regression analysis

**Fig. 2:**
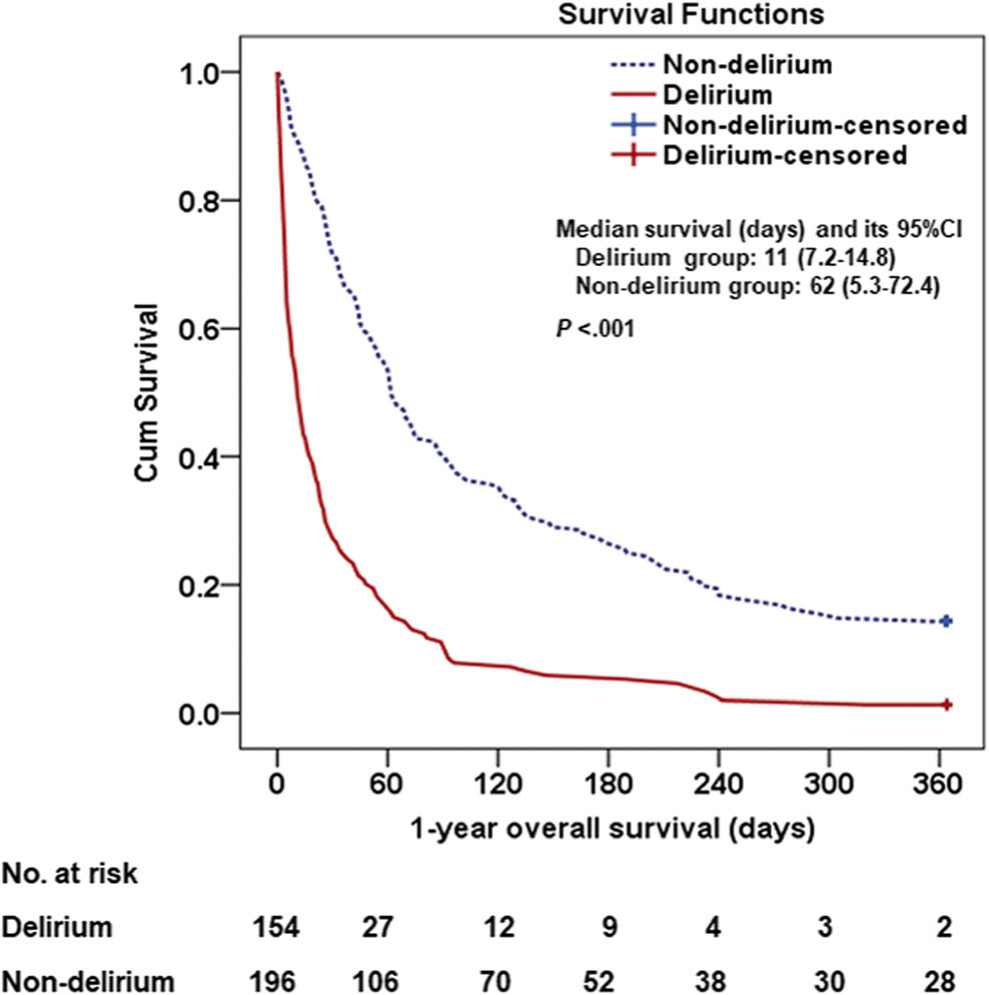
Kaplan–Meier curves of 1-year overall survival of patients referred to palliative care center in delirium and non-delirium groups (Log-rank test *P* < .001)

The median overall survival time was 34 days (95% CI, 25.7–42.2 days), while the overall survival rate was 8.6%. The median overall survival time and overall survival rate of the delirium group was 11 days (95% CI, 7.2–14.8 days) and 1.3%, respectively, compared with 62 days (95% CI, 5.3–72.4 days) and 14.3% for the non-delirium group. The survival data of the delirium and non-delirium groups were compared using a log-rank test, which revealed a significantly higher survival time for the non-delirium group (*P* < 0.001; Fig. 2).

For 210 patients in the IPD group, 93 (44.3%) patients had no delirium, 28 (13.3%) had a correct diagnosis of delirium, 89 (42.4%) had a missed delirium diagnosis, and no patient had an incorrect diagnosis. In other words, of the total of 117 delirious IPD patients, the PMTs had missed the delirium diagnosis in 76.1% (89/117) and made a correct diagnosis for 23.9% (28/117).

## Discussion

To our knowledge, this is the first study to examine the overall prevalence of delirium in newly referred palliative care patients in both OPD and IPD settings simultaneously [4, 25]. We found the overall prevalence was as high as 44%, with the IPD patients having a higher rate than the OPD patients. It is known that IPD patients tend to be in the later stages of disease and have more severe medical conditions, resulting in a higher prevalence of delirium. Our research also found that the prevalence of delirium increased as death approached, with 88.9% of patients being delirious on their last few days of life. Other studies have similarly reported that terminal delirium occurred in 88–93% of patients [2, 18, 26]. This remarkably high prevalence rate underscores the need for discussions to be conducted as early as possible about the goals of care, advance care planning, and the need to take care of unfinished business. Mixed delirium was the most prevalent subtype in our population, followed by hypoactive and hyperactive delirium; this concurs with most of the previous studies [6, 27–29].

While the univariable analysis identified 18 factors associated with delirium, the final model only utilized 8 variables. We confirmed that older age, a lower performance status, and brain metastasis were independent predisposing factors associated with delirium, which is consistent with the findings of earlier work [15–17, 30, 31]. Urinary tract infection, sepsis, and hyponatremia were independently associated precipitating factors of delirium [30, 32–34]. While hypercalcemia was also an independent associated factor, its OR was not able to be calculated because there was no event in the non-delirium group. We found that nearly one-fifth of the delirious patients had hypercalcemia; on the other hand, there were no hypercalcemic patients without delirium. As hypercalcemia is a correctable precipitating cause of delirium [31], we suggest that serum calcium should be checked in delirious patients. In addition, a considerable number of patients did not have any apparent precipitating cause. Such patients should still be promptly treated for their delirium symptoms, and they might need palliative sedation in cases of refractory agitated delirium [35, 36].

Interestingly, in contrast to most of the previous studies [18, 19, 26, 30], we found that psychoactive medications were not associated with delirium. This may be because our study recruited only newly referred palliative care patients, for whom the median morphine equivalent daily dose was only 11.25 mg. There has been reported to be a decreasing trend in the dosage of opioids prescribed for referred palliative care patients [37, 38]. One study reported that cognitive dysfunction was associated with opioid doses exceeding a 400 mg morphine equivalent daily dose, which is far greater than the amount used for our population [39]. Benzodiazepines were also not associated with delirium for the same reason.

As with previous studies [18, 40], our results showed that patients in the delirium group had a shorter survival time (median: 11 days) than those in the non-delirium group. This highlights that delirium is a very poor prognostic factor. However, in contrast with previous studies, the patients in the delirium group had a shorter length of stay than those in the non-delirium group [8, 41]. This may be because the delirium patients in our study had a shorter survival time and, thus, a shorter length of stay, than the non-delirium patients.

We found that the PMTs missed delirium diagnoses in most of the cases (76.1%). This level is similar to the findings of previous studies that investigated the degree to which delirium diagnoses were missed [10, 42]. The data confirms that delirium assessments are still underperformed and that measures to raise awareness of this condition are needed.

### Clinical implications

Delirium is highly prevalent in newly referred palliative care population, causes substantial distress to patients and their families, and significantly associated with shorter survival. Therefore, delirium screening in this population needs to be emphasized. Multiple predisposing and precipitating factors of delirium in this population were identified. This could facilitate in proper investigations and timely interventions.

### Study limitations

There are some limitations to our study. For one thing, we excluded patients with persistent symptoms such as pain, dyspnea, and depression. As it is possible that those patients might have had high symptom expression due to delirium, excluding them may have resulted in a lower prevalence rate than otherwise. However, in our usual clinical assessments of patients with a high physical symptom score, we routinely give an instant treatment such as an oxygen supplement or immediate — release opioids. This routine procedure resulted in only 63 patients out of 743 (8.48%) being excluded, which we believe would not lead to a significant selection bias. Another concern is that we excluded 10 patients with dementia, due to the complexity on the diagnosis of delirium on top of dementia. Nevertheless, this small number should neither affect the overall prevalence rate nor association with other factors. The results of this research might therefore not be applicable to dementia patients. Lastly, the refusal rate of participation in this study was quite high (254/743, 34.19%). However, in the recruitment process, ineligible patients were all excluded before invitation so this also would not lead to a significant selection bias. Most of them just refused because of time constraints.

## Conclusions

The overall prevalence of delirium among the referred palliative care patients was as high as 44%, and the rate rose as death drew nearer. Older age, a lower PPS, brain metastasis, urinary tract infection, sepsis, hyponatremia, and hypercalcemia were independent factors associated with delirium. Delirium was significantly associated with a shorter survival, with a median survival time of only 11 days. Despite the high delirium prevalence rate and the condition being a very poor prognostic factor, there was still a markedly high rate of missed diagnoses of delirium. Effective, routine, delirium screening of palliative care patients needs to be given much greater emphasis.

## Data Availability

The data that support the findings of this study are available on request from the corresponding author, ST. The data are not publicly available due to their containing information that could compromise the privacy of research participants.
